# Past, Present and Future: Perspectives on an Oral History of Intellectual Disability Nursing

**DOI:** 10.1177/17446295211065195

**Published:** 2022-02-10

**Authors:** Carmel Doyle, Colin Griffiths, Su McAnelly, Helen Atherton, Michelle Cleary, Sandra Fleming, Bob Gates, Paul Keenan, Paul Sutton

**Affiliations:** School of Nursing and Midwifery, 155263 Trinity College, Dublin, Ireland; School of Nursing and Midwifery, 155263 Trinity College, Dublin, Ireland; Nursing, Midwifery and Health, 5995 Northumbria University, Newcastle upon Tyne, UK; Academic Unit of Adult, Child and Mental Health Nursing, 4468 University of Leeds, Leeds, UK; Centre of Education and Training, 63974 Muiriosa Foundation, Monasterevin, Ireland; School of Nursing and Midwifery, 155263 Trinity College, Dublin, Ireland; College of Nursing, Midwifery and Healthcare, 7364 University of West London, London, UK; School of Nursing and Midwifery, 155263 Trinity College, Dublin, Ireland; College of Nursing, Midwifery and Healthcare, 7364 University of West London, London, UK

**Keywords:** oral history, intellectual disabilities, nursing

## Abstract

Thirty-one participants engaged in this oral history research study aimed at exploring the lived experience of intellectual disability nurses and healthcare assistants’ knowledge of the trajectory of intellectual disability nursing over the last 30 years in the Republic of Ireland and England. This paper documents some of these experiences offering perspectives on intellectual disability nursing and what is important for the future. Findings from Ireland consider the nature of intellectual disability services and the registered nurse in intellectual disability. Findings from England focus on opportunities and restrictions in intellectual disability nursing, shared visions, the changing context within which work took place and also the internal and external supports that impacted their roles. It is evident that intellectual disability nurses must be responsive to the changing landscape of service provision and also the requirements for contemporary new roles to meet the changing needs of people with intellectual disabilities.

## Introduction

As a specialist part of the wider healthcare professions, intellectual disability nursing has been maintained and endorsed by many as unique in its breadth of employment base, this being located among diverse sectors and service providers (Gates, 2011; McCarron et al., 2018). This distinctiveness makes it valued for the contribution it makes to the lives of individuals with intellectual disabilities. Intellectual disability nurses provide support across multiple residential and community settings. Current practice in intellectual disability nursing has also seen the development of new specialist positions such as community support specialists, liaison posts, epilepsy specialists, as well as offering a range of specialisms in more generic community nurse roles, with these roles offering support across the age continuum (Gates & Mafuba, 2015; Manthorpe et al., 2004; McCarron et al., 2018).

However, despite the specialist nature of intellectual disability nursing and the known contribution it makes to the lives of individuals with intellectual disabilities and their families (Doody et al., 2017; Brown et al., 2016), the numbers of intellectual disability nurses remain relatively low. In the United Kingdom (UK), the numbers of registered intellectual disability nurses have continued to fall from 18,546 in 2015 to 17,125 in 2019 representing an 8% reduction (Nursing and Midwifery Council (NMC), 2019). In the Republic of Ireland, the numbers of registered intellectual disability nurses have also fallen from 6,085 in 2016 to 5,180 in January 2019 representing a 15% reduction (Nursing and Midwifery Board of Ireland and An Bord Altranais, 2020). Over the past 20 years, shortages of intellectual disability nurses have resulted in service providers employing general and children’s nurses to deliver care and support rather than intellectual disability nurses (McGonagle et al., 2004); some of these from countries such as India and the Philippines, who were nurses predominantly trained in the areas of general, mental health and childrens nursing (Humphries et al., 2012; McGonagle et al., 2004). Additionally, these shortages have led to the development of new posts for healthcare assistants and an emerging workforce of social care workers to meet the social care needs of those residing in residential care (Health Service Executive, 2011; Keenan, 2017).

While deinstutionalisation took place earlier in England compared to the Republic of Ireland, currently contemporary models of service for individuals with intellectual disabilities envisage individuals living in their own homes or smaller community settings with a shift away from congregated settings. This is happening somewhat slower in the Irish setting than in the UK (Gates et al., 2020). Over the last three decades, intellectual disability nursing has progressed from the narrowly defined roles it occupied within long-term institutional care services to broader roles within a complex landscape of health and social care service provision. Intellectual disability nurses in the Republic of Ireland have developed their inter-professional practice through leading and co-ordinating effective care for the benefit of individuals with intellectual disabilities (McCarron et al., 2018). Notwithstanding such developments intellectual disability nursing numerically remains one of the smallest of the four disciplines of nursing practice in both Ireland and England. However, the implementation of national policies affecting the lives of those with intellectual disabilities still requires intellectual disability nurses (Health Education England, 2018; McCarron et al., 2018; Merrifield, 2018; National Health Service, 2018). This ongoing need for intellectual disability nurses suggests that they are still considered essential contributors to the lives of individuals with intellectual disabilities. Therefore, it is important to document how intellectual disability nursing has grown and changed over the years in order to better understand how nurses can best support individuals with intellectual disability in the 21st century. Accordingly, this article documents some of the experiences intellectual disability nurses and healthcare assistants have had over the past 30 years with a view to understanding perspectives on intellectual disability nursing and how the profession might develop in the future.

## Aim of Study

The aim of this study was to explore the lived experience of intellectual disability nurses and healthcare assistants’ knowledge of the trajectory of intellectual disability nursing over the last 30 years in the jurisdictions of the Republic of Ireland and England. This incorporated the examination of factors affecting the sustainability of this workforce and how it relates to the contemporary issues of recruitment and retention. Furthermore, an archive of intellectual disability nurses’ and healthcare assistants’ oral histories was established as part of the project.

## Methods

This qualitative research study was underpinned by an interpretive style, phenomenological in nature and undertaken by adopting an oral history approach. Oral history as a method in nursing research has been acknowledged as important (Thomas & Rosser, 2017), recognising the personal narrative as central to eliciting meanings that are useful in revealing past experiences. It has been used as a tool to recall events for many centuries (Atkinson, 2012). With this in mind, it was important to learn from the past in order to inform present and future practice. This approach is not unprecedented in the discipline of nursing (Thomas & Rosser, 2017). However, examples of oral history research in the field of intellectual disability nursing are rare, but there are studies that have sought to catalogue events, just not using an oral history approach (Mitchell, 1996; Nehring, 1999; Sweeney, 2003). The oral history research approach utilised in this unique Anglo-Irish study offered a means of capturing the views and lived experiences of individual nurses and healthcare assistants in two jurisdictions. In effecting this research project, the work has been bench marked against the ‘*Oral History-Good-practice Guidelines’* published by the Heritage Lottery fund (Heritage Lottery Fund, 2014).

### Ethical Considerations

Ethical approval was received across both jurisdictions for all sites both through the X and X (names of Universities). All participants provided written and verbal informed consent prior to and during the interview process. Furthermore, nursing participants who agreed to have their anonymised transcript and audio-recording archived in the Royal College of Nursing (RCN) archives signed the copyright assignment forms. Each participant was assigned a participant identifier number (PIN) starting at 001 and suffixed with either ‘UKE’ or ‘IR’ depending on which jurisdiction they were from. Access to data was restricted to research team members only and was password protected.

### Recruitment

In England, snowball sampling was adopted to access the sample with the researcher identifying one or more individuals from the population of interest. Using Robson’s (2016) guidance, following interview, individuals would identify other members of the population, who are themselves then used as informants and so on. In Ireland, purposeful sampling was used with potential participants from two intellectual disability service providers invited to participate through the medium of gatekeepers who were appointed in each service.

### Inclusion Criteria

Specific inclusion criteria was adopted: ‘*Registered Intellectual Disability Nurses, including State Enrolled Nurses (SENs) from the Republic of Ireland and the United Kingdom, along with HealthCare Assistants, from both jurisdictions who had worked for 30 years or more in the profession, and who had been employed for 30 years or more in congregated or semi-congregated settings, for example, group homes/hostels/hospitals with a minimum of six service users’.*

In order to generate interest from potential participants the project was initially promoted through the intranet pages of both Universities, as well as promotional pieces in both the *Nursing Times* (Merrifield, 2018) and *Learning Disability Practice* (Walker, 2018); both well-known nursing journals, viewed by many practitioners and students of intellectual disability nursing. A total of 31 participants were recruited to the study, 11 to the Republic of Ireland arm of the study and 20 participants to the English arm of the study (Table 1), with length of service anything from 31 to 47* *years.

**Table 1.** Participant details.

	Nurse	Healthcare assistant	Gender	Total
UK	18	2	14 female	20
			6 male	
Ireland	10	1	10 female	11
			1 male	

### Data Collection

Semi-structured interviews were used for data collection. In both jurisdictions, the procedures adopted were broadly comparable; once a possible participant became known, they were initially contacted through email with an attached letter and participant information sheet. If they replied confirming their interest, they were asked to supply a telephone number in order that a telephone call could be made at a mutually agreeable time to further explain the project and establish a possible interview date and venue. Prior to the interviews, all potential participants were posted a covering letter, a participant information sheet, two informed consent forms (one to be retained by the participant), a copyright assignment form (for the RCN archives) and finally a participant diary. The diary was used as a means of enabling each participant, should they choose to use it to write down and detail their ideas and memories from the history of their working lives. It was suggested that they could highlight those parts of their experiences that they considered to be of most interest and they could use this as a reference guide when interviewed. The diaries were held by participants and were not analysed as part of the research. Each participant was then interviewed on the agreed date and venue. Prior to interview, the project was further clarified to each participant before seeking written and informed consent and copyright assignment. After each participant had been interviewed researchers collected a self-disclosed data information sheet from them in order for biographical data to be collected on the participants.

### Data Analysis

All interviews were transcribed verbatim. An overall approach was agreed by the research teams in both the Republic of Ireland and England. The process of data analysis itself adopted the use of a framework of thematic data analysis (Thomas, 2006). This approach involves the identification and labelling of specific text segments to determine codes. These codes were then subsequently reduced through the identification of any overlap and redundancy of text within the codes. The final stages involved clustering the codes into categories, and then grouping these categories into themes. Furthermore, the process of analysis was conducted independently in the Republic of Ireland and in England in order to allow for similarities and differences within the texts to emerge. At key stages in the analysis, the research teams in both the Republic of Ireland and England employed a triangulated approach with other members of the team to consider emerging themes from the data within the context of the key aims of the project. The result was a complex, sometimes similar and at other times contrasting mesh of themes which shed light on the issues in question. Additionally, for those research participants who agreed, arrangements were made for digital recordings of their oral histories to be placed in an online archive held by the RCN, United Kingdom (Republic of Ireland *n* = 8, England *n* = 20). A rigorous research process was upheld throughout, aligned with the criteria propounded by Lincoln & Guba (1985). Peer scrutiny, reflective commentary and participant checks on transcripts were undertaken. At all times, each element of the research process was transparent and an audit trail of decisions made evident.

### Findings

Because the process of analysis took place independently within the Republic of Ireland and England, findings are reported separately from each jurisdiction and discussed jointly thereafter. The resultant emerging themes align with the aim of the study, to explore the lived experience of intellectual disability nurses and healthcare assistants as well as outlining the evolutionary trajectory that intellectual disability nursing has undergone over a thirty year period.

### Republic of Ireland

In thematic analysis of the findings from Republic of Ireland, four overarching themes emerged, the nature of intellectual disability services, the nature of the Registered Nurse in Intellectual Disability (RNID), lifetimes journey and societal change (Table 2).

**Table 2.** Summary of themes for both jurisdictions.

England	Republic of Ireland
Shared visions and shared agency	The nature of intellectual disability services
Opportunities and restrictions	The Nature of the Registered Nurse for Intellectual Disability (RNID)
Internal and external support	Lifetimes journey
Changing contexts	Societal change

### The Nature of Intellectual Disability Services

This theme was concerned with the nature of the services in which participants had worked over the past 30–40 years and how they had changed over time. One of the points raised was that small organisations are better suited to delivering appropriate services to individuals with an intellectual disability than large ones. A degree of disenchantment was articulated regarding the way things had been in the large institutions of 30 or more years ago. Shared clothing, the constant presence of large groups and absence of privacy were some of the features of care recalled by participants.

*(name of unit) unit which was an elderly unit and I suppose there would have been 30/33 residents there and I suppose they had dormitory type settings. (IR6,* Gates et al., 2020. 91*)*

The fittings and furnishings of living places were standardised and more suited to a hospital rather than being places to live.

…before they all had just these little steels beds you know now they have these state of the art hospital beds or they have a double bed. (IR9)

Activities were not organised individually but the whole unit would do things together; …like years ago we say in the summer (we are after having the good weather) you would pack up your 23 clients, you will fill laundry bags with blankets and bottles of miwadi (orange drink) and plastic cups and you would head off down the field. And you would stay there all day somebody would come up and bring the lunch down and you would stay there all day and they loved it, everybody go home absolutely shattered. (IR9).

However, services varied according to who ran them and the degree of disability of the service users. Over the years things changed, especially new staff who had not worked in campus-based congregated settings.

…they’d be more outgoing, less restriction, less kind of barriers as such. Like you know game to try things, to do stuff, see the person as a person. (IR5)

Overall, criticisms of contemporary services largely related to the amount of paperwork and the difficulties of keeping up with the Health Information and Quality Authority (HIQA) inspections. On the plus side, participants referred to the involvement of families and the person being involved in decision making.

### The Nature of the Registered Intellectual Disability Nurse

The values associated with intellectual disability nurses were clear within this theme and it suggests that dedication to each individual with intellectual disability is one key to the sustainability of the nursing workforce. At the outset, one participant stated a fundamental point regarding intellectual disability nursing; I had no brief as to what a good quality of life was for people, I had no yard stick. (IR1)

In other words intellectual disability nursing had no theoretical or knowledge base, the nurse was working in a vacuum. Subsequently, the same participant had to work out the answers to care herself, for example, understanding the cognition of the users of her service.

I suppose for me one of the big things would be trying to put myself into their shoes and to see how life looks like from that individual’s perspective or whatever. And trying to see you know what can you do different or what would you do to help them. (IR5)

A sense of a strong dedication to the individual with intellectual disability emerges from these participants. This implies that nursing is about finding solutions with and for individuals. However, nursing is not so simple and ‘*it is not an easy job’* (IR2). Others noted the challenge of managing staff and not having sufficient resources. Managing those individuals with behaviours that challenge was noted as being particularly difficult highlighting that intellectual disability nursing was regarded as rewarding but hard at times. Another important aspect of being an intellectual disability nurse was the stigma attached to the job. Many of the participants described other nurses referring to them in a patronising manner and indeed some had a sense that they were not regarded as ‘proper nurses’.

So they came with a background of the real medical model of nursing. Whereas we did not and I kind of did feel alright that when we arrived on a ward it was like ah, and we did hear terms like the Fisher Price nurses and that sort of stuff. (IR5)

### Lifetimes Journey

This theme was characterised by participants’ memories of the trajectory that their lives had taken and the changes that were witnessed through the years, opening with their reminiscences about how they got involved with nursing. The emotional commitment that was part of training in this discipline of nursing was evident.

I remember there was a little girl and she was different to the rest and I felt sorry for her and I said oh, I’m going to mind her and I said Oh My God, this is amazing, wouldn’t I love to be minding somebody like this. (IR8)

Many of the participants noted the influence of the Roman Catholic church on their interest in nursing; however, some had entered the profession through extra-curricular activities in school.

Well back in 1971 I was involved in a schools project with the local community parish for people with disability. (IR4)

For others, career guidance teachers steered them towards intellectual disability nursing. However, what most had in common was that they had met or knew individuals with an intellectual disability. On entering the profession as students, many felt out of their depth. Bearing in mind that service provision was campus based and that each residential unit in most campuses consisted of 10–20 or more residents all living in one place these could be bewildering establishments.

*I couldn’t understand where did all these people this big huge day rooms maybe twenty people in the them and them all marching around walking around and I just couldn’t get my head around it for an awful long time... (IR2,* Gates et al., 2020. 101) *I was put into one of these big rooms with all these people on my own and I was standing there at 18 years of age and I thought what have I got myself into? (IR2)*

Once settled in, training was well regarded by most participants.

‘our years of training … they were like so wonderful because you were doing the work on the ground and then you were going back to the books, and I still to this day feel that is the most amazing way to learn’. (IR1)

A general sense of love and care permeated the comments of the participants.

…you were looking at wonderful people with the most amazing personalities, even the most profound (ly intellectually disabled) had amazing personality …and love to be got from you and to be given to you. (IR1)

Many characteristics of what a long professional life in the field felt like were described, however, an outstanding positive was the notion of teamwork:

I’d say my biggest support is from my colleagues. I have a very good team. (IR7)

Also notable was that most participants felt they had lived a meaningful working life.

I’ve enjoyed or still do enjoy the work that I do and I can’t see myself anywhere else really. (IR5)

### Societal Change

Societal perspectives of intellectual disability have changed over the past 30 years or so and contemporary changes in service provision reflect this. The most notable change has been the exodus from congregated settings and participants acknowledged they have been integral to that process.

I think we have come a long way in trying to get it right for the person that’s living in the community. (IR6)

This was further explained by a participant who is currently involved in the deinstitutionalisation process who described it as:

now we’re coming on to the next stage where we’re now going out to community houses where it will be like a home. (IR8)

This trend in society has been generated and reflected by a change of values in society and in the ethos practised by intellectual disability nurses.

*but no I think a lot of the young people now they believe the values, they believe that these people have rights, I really think we talked about it but never believed. (IR1,* Gates et al., 2020. 112*)*

There is a requirement for the nurse to see each individual with intellectual disability on their terms and to try their best to understand them in order to develop approaches to care and support that work for the individual. This was summed up by one participant who while reflecting on how her thinking had changed some 15 years ago stated:

It was a big turning point for me when I see that the (challenging) behaviour was a form of communication not as a result of the condition. (IR7)

### England

In thematic analysis of the findings from England four overarching themes emerged, shared visions and agency, opportunities and restrictions, internal and external support and changing contexts (Table 2).

### Shared Visions and Shared Agency

The first theme explored participants’ views on how and why they entered the profession of intellectual disability nursing and some of the issues that occurred to them as being important in fostering a long-term career in nursing. This was important in understanding the contemporary issues of recruitment and retention. The category ‘sense of justice – doing the right thing and making a difference’ was a powerful statement that many participants felt that was important to their understanding of how their working life had offered them meaning.

…It’s what makes a difference to people in their homes, in the community or wherever they might be and that is really rewarding. When you can see the journey that people go on because of the trauma in people with learning disabilities lives is phenomenal. Every case you read is all about trauma and abuse and neglect, disadvantaged upbringings and then you can see them kind of accept that they’ve made some mistakes along the way but to start a new recovery path and just to have a much better quality of life and feel much better about themselves. (UKE5)

Many participants pursued intellectual disability nursing early on in their lives after meeting someone who had an intellectual disability. And indeed the vision and passion expressed in these interviews to some extent was derived from those early encounters. Much was recalled and shared regarding the enjoyment and the passion that nurses felt as they reflected on the long years of their careers.

…I always enjoyed the work, I knew lots of people, it was an absolutely amazing friendly place to work, we had our own staff club, we had our own big canteen, everybody knew everybody….I love my job still…I liked the work, I liked the people that I worked with, staff and service users. We were a family I think in the old days. It still is in certain areas now. (UKE3)

In general, the participants indicated that they had enjoyed their work deeply and passionately and looked back very fondly on the past, intimating, they would ‘do it all over again’.

The bit that I enjoyed the most must have been my first ward that I was Sister on...I grew up there …I was only…not 23 when I was Sister so …Very very young so I grew up with the old chaps you know and I…sorry I’m going to cry….I absolutely adored it. I just…love that experience and I think something of me got lost when I had to move from there but we move on …but that experience was just one …I certainly recall people and events that they move me as well. I look back and think I wonder what happened to…Yeah I mean I followed them up for a lot of years, as long as I could but a lot of them died. They were in their 90’s, 80’s/90’s… (UKE2)

However, many experienced severe stress which for some developed into burnout. These stressful episodes are conceptually linked to the resilience and coping mechanisms that the participants developed in surviving the many years of practice. For many participants, surviving and coping largely related to the supports they received. Furthermore, staying in the profession for some participants resulted in experiencing significant structural changes in the way they worked over the years.

### Opportunities and Restrictions

The changing personal and professional landscape over the years required nurses to adapt to the new opportunities that evolved.

…*Then I did a Certificate, that’s what it was called, and it was a Certificate of Forensic Issues I think it was called the course I did. So I did that and then after a few months of finishing that, completing it, I still thought I need more. It really made me think about things. (UKE3)*

Many female participants who entered the workforce without any educational qualifications recounted how they studied to become registered nurses and then subsequently took up specialised post graduate diploma and masters courses.

So I did the Diploma in Community Nursing and then in 2005 I did my Nursing Degree with the RCN …and got a 2:1 which I was quite pleased about really given the fact that I was still working full-time and had a family. (UKE10)

On the whole, education was seen by participants as the key to developing new knowledge and skills. However, some male participants reported that they achieved senior positions quite quickly after training either because of their gender or because of their physical size and capabilities which were useful in the institutions of that time.

*I said well if you make me a Deputy Charge Nurse and give me a staff house I’ll stop and he did! So I wasn’t married, he gave me a three bedroom terraced house, staff house and he made me up to Deputy Charge Nurse. So I was never a Staff Nurse. (UKE7,* Gates et al., 2020. 77*)*

Nearly all participants thought of the intellectual disability nurse as being undervalued by other healthcare professionals and this had important consequences for how they shared their vision of their work with others. This participant had a strong visceral memory of such an incident.

I’ve even had somebody say to me I can smell that hospital on you, on your clothes… As if repulsed you know, as if repulsed. (UKE4)

### Internal and External Support

Team working was considered to be very important by participants and could be broken down into working both in formal and informal teams. Team working was also considered to be imperative to enabling the participants to be resilient to the pressures of the job and to facilitate them to attain the shared vision for individuals with intellectual disability whom they supported. Support from other professionals and from family members was important to allow participants to engage in the long haul of working over 30 years or more in the profession.

Love it! It’s massively important, you’re nothing if you’re not a team. Nothing! And leading teams, or members of teams, everybody is the same, everybody has the same value, you’re not more important or less important than anybody else. I love that…that’s the best…apart from service users, that’s the next best thing. I love being in teams. (UKE5)

By contrast professional bodies (such as the NMC and the RCN) and other external agencies were not thought to have impacted on the nurses’ careers or the ability to cope with professional challenges.

### Changing Contexts

Participants felt strongly about how practice had changed over the past 30 years. There was no suggestion of a faultless past, however, there was a sense that the essential values that participants had cultivated long ago were important and that perhaps some of these had gone missing during the move from institutions to community.

…we used to have those meetings where you could talk about your work and talk about things that were difficult. You’ve got a different disciplines point of view. It felt very supportive and that’s missing. (UKE4)

Nearly everybody felt that Government and particularly the financial aspects of governance greatly impacted the ability of the nursing community to do its job. It was felt that something of importance had been lost. And this emotion was connected to the idealism of the participants starting out on their careers in order to make the world a better place.

…In the old days we used to go out on lots of trips with them, take them out and do things, like I say sit and have a brew with them on the ward. (UKE3)

Many nurses felt that in the distant past, their learning or at least some part of it was achieved through negative experiences, that is, identifying what was not good in practice and changing it. The 1980s and 1990s were noted as a time when education and leadership in the field challenged nurses to lead change. Participants accept that the world is now a different place particularly in terms of safeguarding vulnerable people, however, they felt that professional distancing instituted a barrier between relationships and placed nurses in a very formal role, something different to the one they had known at the start of their careers. Overall, these participants reflected back on their professional lives with fondness, great clarity and a sense those were days that would never return.

*My experience was very good levels of staff. But then again we got them* (the service users) *out every day. I felt I had a cushy number actually because what could be nicer than walking in the county area? (*UKE1)

## Discussion

This discussion intends to examine the central underlying issues that nurses in both jurisdictions were concerned with. The aim of the study, to explore the lived experiences of intellectual disability nurses and healthcare assistants is to the forefront in discussion. It also examines the core worth of the intellectual disability nurse and what new perspectives can be envisioned. Despite intellectual disability nurses being the only professional group specifically trained to work with individuals with intellectual disabilities, this research reported that those from both jurisdictions felt undervalued by their colleagues in the nursing profession and also by wider society. There was a sense that the nursing profession as a whole regarded them as ‘half nurses’ and the stigma which was acknowledged attaches to individuals with intellectual disability also attaches to those who care and support them. This undervaluing by others raises the question of what is the value of the intellectual disability nurse for individuals with intellectual disability? And by implication does the nurse who works with individuals with intellectual disability offer something that others cannot? Also, of importance, is the question of how individuals with intellectual disability view intellectual disability nurses? It is acknowledged that there is lack of understanding by other nursing disciplines, health professionals and the wider public about what intellectual disabilities is and moreover what the intellectual disability nurse does (Department of Health and Children, 1998; Owen and Standen, 2007; While and Clarke, 2010). Historically, the attitudes of general nurses (Lewis and Stenfert-Kroese, 2010) and senior psychiatry doctors (Ouellette-Kuntz et al., 2003) towards individuals with intellectual disabilities have been identified as being negative at times. General nurses often feel insufficiently and ineffectively prepared to support individuals with intellectual disabilities (Applegren et al., 2018), something that could be ameliorated by referring to the intellectual disability nurse. In recent years, this has been somewhat addressed by the development of acute liaison nursing roles for intellectual disability nurses working to support individuals with intellectual disabilities in acute settings (Brown, 2020). Consideration of the significance of intellectual disability nursing in supporting individuals with an intellectual disability is crucial in ensuring sustainability of the profession. Without value placed on the role, recruitment into this field would be difficult.

Of note in considering the values reported by the participants in this project is the commitment that they made to those whom they cared for. Participants reported having a vision of what they wanted for individuals with an intellectual disability whom they cared for. Moreover, they also thought that it was important to do the right thing in their work and to do their work with passion. Moreover, they did not just go to work out of routine but out of commitment, almost discerning a vocational aspect to their attitude to their work. Martin et al. (2012) remark on the emotional and personal commitment to the people whom they serve that intellectual disability nurses report. Their participants mostly exuded a feeling that they loved their work, despite certain downsides to it. It was noted that as participants gained more experience, their confidence grew accordingly, something that is an important element of intellectual disability nurse education across the lifespan (; McCarron et al., 2018; Northway et al., 2017). It is also arguable that emotional and personal statisfaction in the workplace is a powerful motivator that works as a strong driver of staff retention and should therefore be valued and promoted by health service employers.

It should also be noted that this was an enduring commitment, something that lasted for most of these respondents over the whole of their careers which lasted 30 or more years; a remarkable achievement. All participants in this study had by definition worked for at least 30 years with individuals who have an intellectual disability so by implication each participant had demonstrated unusual resilience in their career. The Irish participants noted that at times the job was a difficult one and that service users could present with challenges, not to mention the periodic difficulties presented by staff and under resourced systems**.** Similarly, the English participants noted that burnout was an ever-present risk. One difference between the jurisdictions was that the framework for delivery of care and support changed much more and faster over the years in England and therefore, navigating the dynamic background presented an ongoing challenge. Resilience comes with connotations of toughness, an ability to undergo and come through adversity as an enhanced individual and many of the participants in both jurisdictions appeared to share these characteristics. Again, this type of resilience is important in ensuring a commitment to the field and the maintenance and development of a sustainable workforce to support individuals with intellectual disabilities.

Yet, various issues have and continue to impact intellectual disability nursing. Of primary importance is the context in which it takes place where nurses have to balance a ‘nursing’ or healthcare oriented philosophy with the social model of care provision which is essentially rights based and far more assertive of the rights of the person (Doody et al., 2019). Over the years, there was a continuous shift in the nature of service provision. In order to meet the reorientation of nursing that focused on the needs of the individual in the context of society rather than in the context of an institution, the education trajectory of intellectual disability nurses through undergraduate, post graduate and other courses continually developed to meet the demands of this changing landscape (McCarron et al., 2018).

Another consideration relates to the intensity with which nurses attempt to comprehend those with severe and complex disabilities. Martin et al. (2012) report that such is the difficulty and complexity of communication with these service users that intellectual disability nursing in this context can be considered a ‘shy’ discipline in that nurses do not articulate their role and work in a strong voice. Presumably, this is because of the complexity and challenge of interpreting others which is so difficult to explain in an unequivocal manner yet is an essential skill for those who support this group of people.

The closing of institutions and the move to more community-based services took place in England earlier and proceeded more rapidly than in Ireland. However, by the late 2010’s most services in both countries were largely community based. Whereas in the UK many services were run by private companies in Ireland this is still a less prevalent type of service. However, for both jurisdictions the underlying culture has changed over the past 30–40 years. Large groups of individuals with intellectual disability no longer live together or partake of life together. Services are essentially oriented around supporting the individual. Equally, the heavily top down quasi-military structures that used to characterise services are gone. No longer do Matrons and Chief Nursing Officers walk the corridors of institutions. Instead, quality is monitored by the Care Quality Commission in England and HIQA in Ireland and this change has impacted on how staff feel about their work and indeed the nature of the work that they do which has become somewhat more bureaucratic. Support has been decentralised to an individual or at least small group level. The values that offer the scaffolding for this approach offer new and developing perspectives and are turning towards the individual taking centre stage and achieving this through attempting to understand how that person views the world and how support can best be deployed to enable the person to achieve the best quality of life possible.

Participants from both jurisdictions emphasised the importance of teamwork and the support that they got from other nurses, professionals and from family. A multi-disciplinary team working environment where intellectual disability nurses would feel supported is a dimension of practice advocated in the literature (Mansell and Harris, 1998; McCarron et al., 2018; Stewart and Todd, 2001). The difficulties that working with others could bring were also noted. Nurses in both jurisdictions regarded building long-term connections between themselves and service users as being important. Good listening skills were noted as being vital particularly when working with service users who were non-verbal. However, nurses also felt that socialising with service users was not part of their brief. A professional distance was emphasised that did not preclude warm, caring relationships but which meant that the relationship was at all times boundaried. Awareness of professional boundaries is alluded to in the literature and the importance of knowing what constitutes over involvement is evident (Bowler & Nash, 2014).

This research revealed the fundamental point that nurses make an incontrovertible contribution to the lives of individuals with an intellectual disability. Furthermore, this contribution occurs across multiple groups of diverse service users and in many different contexts. The recent study exploring the role of intellectual disability nursing in Ireland makes this clear (McCarron et al., 2018) such that some consider the intellectual disability nurse is or should be the ‘go to’ person in the health service. Others have noted how intellectual disability nurses can act as advocates and raise standards in the acute hospital sector (Brown et al., 2016). Indeed, more recently, the provision of expert, complex healthcare is regarded as one of the strong aspects of intellectual disability nurses (O’Reilly et al., 2018). However, the primary argument for specialist nurses is not so much concerned with their skills set or the particular areas in which they apply it, it is more to do with how they work and specifically with how they communicate with those whom they serve and especially those with severe and complex disability who do not verbalise. This key point is emphasised by Jaques et al. (2018) who after reviewing the literature on the contemporary role of the intellectual disability nurse conclude that the skill set of the specialist intellectual disability nurse is not uniquely technical, but is uniquely relational. In other words, specialisation is recognised not in what specialist intellectual disability nurses do, but in how they relate to individuals with intellectual disabilities that they work with. This unique competency involves learning and deploying skills of observation. This requires detailed noting of the expressions and behaviours of people with intellectual disability coupled with close listening, interpreting and then the subsequent analysis of each person’s unique messaging (Martin et al., 2012) with a view to freeing people with an intellectual disability to express who they are in their own way and who they wish to be in all its fullness. This is a complex competency which is difficult to develop but which when it is achieved demonstrates a very high level of skill and reflects the nurse’s potential to unlock the communications of an individual with severe intellectual disability and thereby allow the individual to realise his or her life. Knowledge and awareness of such a potentially profound contribution by intellectual disability nurses to enhance the lives of indviduals with intellectual disability is, we would argue, essential in attracting suitable candidates to enter the profession and should in turn support recruitment and retention of staff.

Service users appreciate intellectual disability nurses for help in accessing and rendering direct personalised healthcare in their home settings (McCarron et al., 2018). Brown et al. (2016) comments that individuals with intellectual disability value nurses for supporting them when they feel vulnerable. Families have noted that they value intellectual disability nurses for support in their caring journey. By implication, nurses are valued as advocates to help access services and to navigate the health service. More generally nurses are seen as facilitating and enabling families and service users to get through life in as meaningful a way as possible. For service users, they are the skilled friend who ‘is always there for us’ (McCarron et al., 2018. 47). Intellectual disability nurses have always played a role in advocating for the individuals they work with. This important role offers a unique support to individuals and their families, one that needs to be sustained into the future.

### Implications for Practice

This study is significant, offering insight into the experiences of years of intellectual disability nursing practice across both the Republic of Ireland and England and the trajectory it took. Awareness of what the intellectual disability role entails needs to be increased to enable appropriate recruitment and retention into the field. In turn, this will enhance sustainability of the profession. It is imperative that intellectual disability nurses are reminded of the relational aspect of their roles. It is expected that they possess key practical skills and competencies. However, the ‘good’ nurse deploys these through an easy, confident and accurate understanding of the person whom he or she is supporting. Practice requires a holistic empathic understanding that will also facilitate the relational aspects of care. Intellectual disability nurses must develop and apply specific approaches to understanding the person with intellectual disability whether working within the confines of a health, social or preferably a biopsychosocial model of care. Furthermore, intellectual disability nurses are advocates for families and individuals with intellectual disabilities and this is one of their most important caring roles. If carried out well it enables families and service users to navigate the system and achieve the highest possible quality of life. This study suggests that there are many different qualities needed to support individuals with intellectual disability. Therefore, recruitment of potential nurses into the discipline requires a broad base of students from all walks of life, backgrounds and ways of thinking, gaining through third level education, academic and practical skills that some may apply for many years, indeed perhaps for a whole lifetime.

## Conclusion

This article has provided insights into the lived experiences of intellectual disability nurses’ and healthcare assistant’s knowledge of the trajectory of intellectual disability nursing over 30 years in the jurisdictions of the Republic of Ireland and England. A number of oral histories have been used to account for and extrapolate key findings that highlight the challenges these intellectual disability nurses have experienced within changing societal contexts and have identified the internal and external supports that were helpful to them. Additionally, the nature of intellectual disability services and the nursing role has been examined. In offering a new perspective on intellectual disability nursing, it is evident that there are key aspects that are important for intellectual disability nurses working with individuals which should include the maintenance of core competencies through continuing professional education. So too must intellectual disability nurses be responsive to the changing landscape of service provision and requirement for contemporary new roles in order to meet the changing needs of individuals with intellectual disabilities. This research contributes to filling a gap in understanding the historical perspectives of intellectual disability nurses and acknowledges the value of the experiences that these nurses have accrued over the years. It also highlights the importance of those experiences as they were shared with people with intellectual disabilities.
